# How Divergence for Feed Efficiency Traits Affects Body Measurements and Metabolites in Blood and Ruminal Parameters on Pre-Weaning Dairy Heifers

**DOI:** 10.3390/ani11123436

**Published:** 2021-12-02

**Authors:** Juliana Mergh Leão, Sandra Gesteira Coelho, Camila Flávia de Assis Lage, Rafael Alves de Azevedo, Juliana Aparecida Mello Lima, Juliana Campos Carneiro, Alexandre Lima Ferreira, Fernanda Samarini Machado, Luiz Gustavo Ribeiro Pereira, Thierry Ribeiro Tomich, Hilton do Carmo Diniz Neto, Mariana Magalhães Campos

**Affiliations:** 1 Department of Animal Science, Veterinary School, Federal University of Minas Gerais, Belo Horizonte 30161-970, MG, Brazil; jumergh@yahoo.com.br (J.M.L.); sandragesteiracoelho@gmail.com (S.G.C.); camilassislage@yahoo.com.br (C.F.d.A.L.); rafaelzooufmg@gmail.com (R.A.d.A.); julianamello85@gmail.com (J.A.M.L.); juccarneiro@yahoo.com.br (J.C.C.); axellfire@hotmail.com (A.L.F.); hiltondinizneto@gmail.com (H.d.C.D.N.); 2 Brazilian Agricultural Research Corporation, Empresa Brasileira de Pesquisa Agropecuária–Embrapa Dairy Cattle, Juiz de Fora 36038-330, MG, Brazil; fernanda.machado@embrapa.br (F.S.M.); luiz.gustavo@embrapa.br (L.G.R.P.); thierry.tomich@embrapa.br (T.R.T.)

**Keywords:** residual growth, residual feed intake, high efficiency, low efficiency

## Abstract

**Simple Summary:**

Improvements in dairy cattle feed efficiency have substantial effects on economic efficiency and can reduce environmental impacts through lower feeding costs and fewer emissions associated with dairy farming. The efficiency of an animal for converting feed into products is influenced by genetic, physiological, and environmental factors that result in individual variations. The utilization of feed efficiency indexes aims to identify and select animals with great economic value in a production system. Associations between morphometric indicators, hormone concentrations, and blood parameters may assist in the identification of differences in the efficiency of feed utilization and in understanding the physiological bases linked to animals’ metabolic responses, thus helping to identify more efficient animals. In our study, it is unlikely that measurements of blood, rumen, or morphometric indicators, per se, will be useful in the early identification of more efficient animals. Understanding the underlying physiological basis for improved feed efficiency in dairy heifers requires further investigation.

**Abstract:**

The objectives of this study were: (1) to evaluate feed efficiency indexes and their relationships with body measurements and blood and ruminal metabolites in the pre-weaning period; (2) to determine if such measurements can be used as feed-efficiency markers during the pre-weaning period. Holstein–Gyr heifer calves (*n* = 36), enrolled between 4 and 12 weeks of age, were classified into two residual feed intake (RFI) and residual body weight gain (RG) groups: high efficiency (HE; RFI, *n* = 10; and RG, *n* = 9), and low efficiency (LE; RFI, *n* = 10; and RG, *n* = 8). Calves were fed whole milk (6 L/day) and solid feed ad libitum. Body developments were measured weekly and feed intake (milk and solid feed) daily during the whole period. Blood samples were collected at 12 weeks of age and analyzed for glucose, insulin and β-hydroxybutyrate (BHB). Samples of ruminal content were collected on the same day and analyzed for pH, NH_3_-N, and volatile fatty acids (VFA). Among the growth characteristics, only the initial hip width differed between the RFI groups, and withers height differed between the RG groups. Concentration of BHB was greater and glucose: insulin ratios tended to be greater in LE-RG animals. Butyric acid proportions were similar among RFI groups, but tended to be greater for HE-RG than for LE-RG. Overall, correlation coefficients between RFI or RG and blood, rumen, or morphometric markers were low. Thus, it is unlikely that measurements of metabolic indicators, per se, will be useful in the early identification of more efficient animals. Understanding the underlying physiological basis for improved feed efficiency in dairy heifers requires further investigation.

## 1. Introduction

Improvements in dairy cattle feed efficiency (FE) have substantial effects on economic efficiency and the reduction of environmental impacts through lower feeding costs and less emissions associated with dairy farming. To increase the biological and economic efficiencies of cattle production, animal selection strategies need to focus on improving FE without compromising performance.

A 5% improvement in FE has an economic impact eight times greater than a 5% increase in average daily gain (ADG) [1]. In addition, breeding for improved residual feed intake (RFI) can enhance feed efficiency without increasing the animal’s mature size [2]. This has obvious positive ramifications for the improvement of the FE of growing and adult cattle. However, there is limited published information on phenotypic RFI and the residual body weight gain (RG) of dairy heifers, as well as on its potential impacts on milk production and the biological factors contributing to variation in these traits.

Residual feed intake is the most-used index of FE [3], and it is defined as the difference between realized and predicted intake, using a linear regression of individual intake as a function of mean metabolic body weight (BW^0.75^) and ADG. This index is independent of growth rate and body weight (BW). Another measurement of efficiency is RG, which is similar to RFI, but instead of regressing feed intake on BW and ADG, as for RFI, RG is obtained from the regression of ADG on feed intake and BW [4].

Since variability in RFI has been acknowledged, there has been abundant research assessing its underlying physiological mechanisms [5,6]. Evidence shows that no single physiological mechanism is responsible for the observed variability [5]. Theoretically, every physiological step that affects the conversion of feed gross energy to animal products could be associated with the observed variability in RFI.

Associations between hormone concentrations and RFI have been studied [7,8], and blood parameters may assist in the identification of differences in the efficiency of feed utilization and in understanding the physiological bases linked to the animal’s metabolic response, thus helping to identify more efficient animals. Circulating the blood metabolites in growing heifers, for example, is responsible for contributing approximately 35% of the variation in RFI [7]. Likewise, studies have been done to evaluate differences in ruminal parameters between high and low RFI animals and their possible role as FE markers. However, there is limited published information on the repeatability of RFI and its associated traits at different phases of the production cycle, which is ultimately essential for wide-scale adoption by producers.

Therefore, the objectives of this study were: (1) to evaluate FE indexes and their relationships with body measurements, blood, and ruminal metabolites in the pre-weaning period; (2) to determine if such measurements can be used as FE markers during the pre-weaning period. Our hypothesis was that there are differences in RFI and RG that explain better feed utilization by some animals, and that such measurements can be used as feed-efficiency markers.

## 2. Materials and Methods

The experiment was conducted at the Embrapa Dairy Cattle Experimental Farm, located in Coronel Pacheco, Minas Gerais, Brazil.

### 2.1. Animals, Housing, Management, and Feed Efficiency Groups

The data presented in this paper is part of a study that classified Holstein × Gyr F1 crossbred heifer calves in high and low efficiency groups using 2 FE indexes (RFI and RG). Detailed descriptions of the methods, performance data, calculation of indexes, and how the animals were classified in high and low efficiency groups are provided in the manuscript [9]. Briefly, 36 heifer calves (BW at birth = 32.4 ± 6.6 kg, mean ± SD) were removed from their dams immediately after birth, weighed, and had their umbilical cords immersed in 10% iodine solution. Colostrum was fed within 6 h of birth (10% of birth BW; >50 g of IgG/L). Passive immunity transfer was assessed using total serum protein. Blood samples were collected via jugular venipuncture within 48 h of birth and centrifuged at 1800× *g* for 10 min at room temperature (22–25 °C), and total serum protein was measured using a refractometer (Serum protein REF-301, Biocotek, Beilun, Ningbo, China).

Heifers were housed in sand-bedded individual pens (1.25 × 1.75 m, tethered with 1.2-m long chains), which were allocated in a barn with open sides and end-walls. Transition milk was fed until 3 days of age, followed by whole milk thereafter until an abrupt weaning at 82 days of age. The volume of milk supplied during the pre-weaning period was fixed at 6 L of milk/day, divided into 2 equal meals of 3 L of milk each, at 0700 and 1400 h. Water and solid feed (starter and chopped hay) were offered in buckets for ad libitum intake (10% orts of solid feed) throughout the experimental period. Solid feed (as-fed) was composed of 95% starter (Soylac Rumen 20% Flocculated, Total Alimentos, Três Corações, Brazil) and 5% chopped Tifton 85 hay (Table 1).

Milk and solid feed were measured between 4 and 12 weeks of age. Feed was weighed using a bench scale (9094 plus, Toledo^®^, São Bernardo do Campo, São Paulo, Brazil.Solid intake was calculated by the difference between offers and refusals measured daily. Daily milk intake was calculated as the sum of the differences between offered and refused amounts at morning and afternoon feedings. Heifers were weighted at birth, at 3 days of age (i.e., when the experimental diet was first offered), and weekly thereafter, always before the morning milk feeding.

The residual feed intake and residual gain were calculated over 56 days of observation. Intake and performance were evaluated from 25 days to 80 days of age. The total DMI was obtained from the sum of milk DMI and solid feed DMI (offered amount minus refusals on DM basis).

The average daily gain was calculated as the linear regression coefficient of BW (PROC REG; SAS Inst. Inc., Cary, NC, USA), composed of nine BW measurements per heifer at 7 days intervals. Metabolic body weight (BW^0.75^) was calculated using the BW at day 23 of the test. Feed efficiency was measured using the relationship between mean daily total DMI and ADG [10].

Linear regressions were used to estimate RFI and RG [3], where RFI and RG were calculated as the difference between realized and predicted total DMI and ADG, respectively, as follows:

Yj = β0 + β1(BW^0.75^j) + β2(ADGj or total DMIj) + ej,

where:

Yj = is the standardized total DMI (RFI) or ADG (RG) of heifer j;

β0 = is the intercept, β1 is the regression coefficient for BW^0.75^;

β2 = is the regression coefficient for ADG (RFI) or total DMI (RG);

ej = is the error term for heifer j.

**Figure 1:**
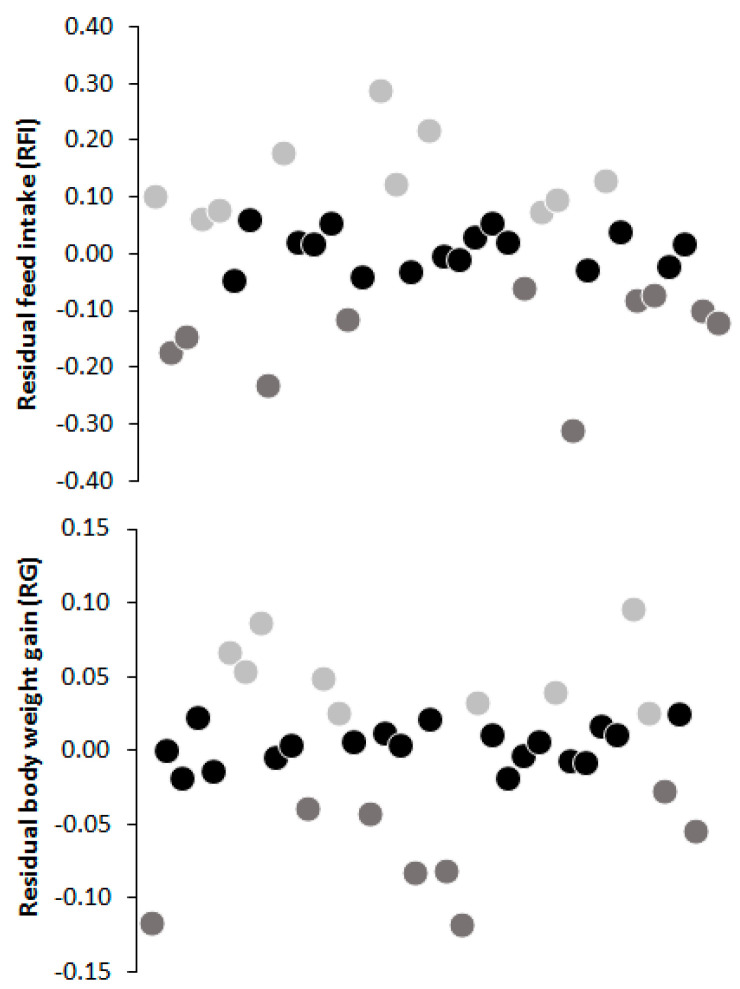
Classification of experimental animals for residual feed intake (RFI) and residual body weight gain (RG) groups: 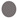 = high efficiency (RFI < 0.5 SD below the mean (n = 10) and RG > 0.5 SD above the mean (n = 9)); 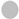 = low efficiency (RFI > 0.5 SD above the mean (n = 10) and RG < 0.5 SD below the mean (n = 8)) and 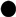 = intermediary.

Heifers were classified into two RFI and RG groups: high efficiency (HE; RFI < 0.5 SD below the mean (*n* = 10) and RG > 0.5 SD above the mean (*n* = 9)), and low efficiency (LE; RFI > 0.5 SD above the mean (*n* = 10) and RG < 0.5 SD below the mean (*n* = 8)) (Figure 1). The remaining animals were classified as intermediate and were not included in subsequent analyses.

### 2.2. Morphometric Measurements

Morphometric measurements were performed weekly (between weeks 4 and 12) before morning milk feeding and after weighing, in a flat location that allowed the animals to remain with their limbs well-set. Withers height (the distance from the withers to the ground) and hip height (the distance from ileosacral tuberosity to the ground) were measured using a hipometer (Walmur, Porto Alegre, Brazil). Hip width (the distance between the two iliac tuberosities), and heart girth (measured immediately and caudally to the front limbs) were evaluated with a measuring tape (Bovitec, São Paulo, Brazil). The variation of each body measurement was calculated as the difference between the final (12th week) and initial (4th week) values.

### 2.3. Blood Collection and Analyses

Blood samples were obtained by jugular puncture 3 h after the morning milk feeding and on the same day of ruminal fluid collection (12th week), during the RFI and RG evaluation period. Samples collected into untreated tubes were used for the dosage of insulin and BHB, and those from sodium fluoride-treated tubes were used to determine glucose concentration (Vacutainer, Becton Dickinson, São Paulo, Brazil). Tubes were placed in crushed ice until centrifugation at 1800× *g* for 10 min at room temperature (22–25 °C). Plasma or serum aliquots were stored at −20 °C until analysis.

Plasma glucose was measured on a microplate Spectrophotometer EON (Biotek Instruments Inc., Winooski, VT) using an enzymatic colorimetric method (Kovalent do Brasil Ltd.a., Rio de Janeiro, Brazil). Serum insulin was analyzed using a bovine ELISA kit (Mercodia, Uppsala, Sweden) and serum BHB was determined using an enzymatic kinetic kit RANBUT—Ref.: RB 1007 (RANDOX Laboratories—Life Sciences Ltd., Crumlin, UK) and spectrophotometry (Automatic System for Biochemistry, Model BIOPLUS BIO 2000^®^, Bioplus Produtos para Laboratórios Ltd.a, Barueri, Brazil).

### 2.4. Rumen Variables and Analyses

Ruminal fluid samples were obtained at the end of the experimental period (12th week). Samples of approximately 50 mL were collected 3 h after milk feeding using a stomach tube technique [11,12,13]. The liquid was double-filtered through cheesecloth, and the pH was measured immediately after collection using a portable potentiometer (DM-2-Digimed, São Paulo, Brazil).

Rumen content samples (5 mL) were acidified with 1 mL of sulfuric acid (500 mL/L) and stored at −20 °C until the analysis of ruminal NH3-N concentration, which was quantified after the distillation of Kjeldahl with magnesium oxide and calcium chloride, according to method 920.03 [14]. Samples were centrifuged at 1800× *g* for 10 min at room temperature (22–25 °C) for the measurement of volatile fatty acids (VFA) concentration by high-performance liquid chromatography (Waters Alliance e2695 Chromatograph, Waters Technologies do Brazil LTDA, Barueri, Brazil).

### 2.5. Statistical Analyses

Data were analyzed using SAS version 9.0 (SAS Institute Inc., Cary, NC, USA). In all models, the normality and homoscedasticity of the standardized residues were evaluated graphically and using the Shapiro–Wilk and Bartlett tests, respectively.

Total DMI, water intake, and performance were analyzed using the linear mixed model (PROC MIXED), including the calf as the random component of the model, and the feed efficiency group, week, and their interaction as the fixed components (efficiency group × week interaction). The birth weight and total serum protein were used as covariates and included in the fixed-effect model.

Variables with a single measurement during the study (blood, rumen, and morphometric variables) were analyzed including the calf as the random term, and the efficiency group as a fixed variable (PROC GLM).

The PROC CORR procedure was used to assess the correlations between the response variables and RFI and RG. For all the variables analyzed, significance was declared when *p* ≤ 0.05, and a tendency declared when 0.05 > *p* ≤ 0.10.

## 3. Results

The residual feed intake ranged from −0.14 to 0.13 kg/day (*p* < 0.01) for HE and LE, respectively, equating to a difference of 0.27 kg of DMI/day between the groups ranked high and low RFI. The residual weight gain ranged from 0.05 to −0.07 (*p* < 0.01), equating to a difference of 0.12 kg of ADG between the groups ranked high and low efficiency for RG.

At the start of the experimental period (week 4), BW ± SD was 46.78 ± 5.81 kg in the RFI test and 45.36 ± 6.98 kg in the RG test. At the end of experimental period (week 12), BW was 101.68 ± 12.89 kg and 99.49 ± 12.68 kg for RFI and RG tests, respectively. The effect of phenotypic classification by RFI and RG on feed intake, feed efficiency, and performance was evaluated in previous work [9], and the data is summarized in Table 2.

Differences were not detected between groups in the RFI test for withers height, hip height, and heart girth (*p* > 0.05; Table 3), and between groups in the RG test for hip height, hip width, and heart girth (*p* > 0.05; Table 3). Among the RFI groups, HE-RFI had greater initial hip width than LE-RFI (22.3 and 20.6 cm, respectively; *p* = 0.03). Moreover, the HE-RG group had greater variation in withers height than LE- RG (15.2 and 12.7 cm, respectively; *p* = 0.01).

Morphometric measurements was not correlated with the feed efficiency indexes (*p* > 0.05; Table 4).

No significant differences in glucose, insulin, BHB concentrations, and glucose:insulin ratios were observed among the groups of RFI (Table 5). For the RG tests, differences were detected between BHB concentrations (*p* = 0.01) and tended for the glucose:insulin ratios (*p* = 0.07). The LE-RG group had greater BHB concentrations than the HE-RG group (0.38 and 0.28 mmol/L, respectively), and a tendency for a higher glucose:insulin ratio (1.69 and 0.83 mg/μU, respectively). There were no significant correlations between blood variables (glucose, insulin, glucose:insulin ratio, and BHB) and RFI, total DMI, and feed efficiency. Significant correlations were observed between blood insulin and glucose concentrations for the RFI (r = 0.46; *p* = 0.05) and RG groups (r = 0.61; *p* = 0.02), respectively. There were negative correlations of −0.54 (*p* = 0.03) between RG and BHB and −0.68 (*p* = 0.01) between RG and the glucose:insulin ratio.

Residual feed intake and RG had no effect (*p* > 0.05) on rumen fermentation (Table 6), except for NH3-N and butyrate concentration. The HE-RG group had greater NH3-N concentrations than LE-RG (10.42 and 6.51 mg/dL, respectively), as well as higher butyrate concentrations (3.99 and 3.05 µmol/mL, respectively). Rumen fermentation variables were not correlated with the feed efficiency indexes (*p* > 0.05).

## 4. Discussion

This study investigated feed efficiency indexes and their relationships with body measurements, blood, and ruminal metabolites during the pre-weaning period, as well as whether such measurements can be used as feed efficiency markers during the pre-weaning period.

The average daily gain and morphometric measurements were similar between HE and LE calves during the RFI and RG test period, similar to those reported by another study [15]. Others have indicated that morphological traits do not differ according to RFI ranking, consequently not altering the morphometric pattern of the animals selected for RFI [7,16,17]. The variation in withers height was greater for HE-RG than for LE-RG, demonstrating a greater growth in the former group. This agrees with results reported by another study [4], which reported high correlations between RG and body growth rate (*r* = 0.70).

In the present study, glucose concentration was not different among RFI-divergent animals. A recent study with pre-weaning dairy heifers also did not observe differences in glucose or insulin between HE and LE RFI groups [18]. In addition, a study with growing Nellore cattle also did not observe differences in glucose concentration between RFI groups, but HE-RFI animals had a lower glucose:insulin ratio and higher insulin concentrations than LE-RFI animals [8]. In this work, the greater satiety of HE-RFI animals was associated with a high concentration of insulin in the blood.

In the present experiment, HE-RG heifers tended to have lower glucose:insulin ratios, indicating a greater secretion of insulin per unit of blood glucose by these animals. Insulin is an important hormonal regulator of the metabolism and an inhibitor of hepatic gluconeogenesis that reduces the hepatic absorption of some glucose precursors, directing the flow of glycogenic nutrients to muscle and adipose tissues [19]. Greater insulin concentrations promote protein and lipid synthesis and body weight gain, which may explain the greater withers height variation in the HE-RG group.

Heifers classified as HE-RG tended to have a lower BHB concentration, but this metabolite was not different among RFI groups. In our study, total DMI did not differ among HE-RG (1535 g/day) and LE-RG (1594 g/day) [9], but HE-RG tended to have greater ruminal butyrate concentrations. In young animals, BHB is produced when rumen butyrate is metabolized by the rumen epithelial cells. The high rumen pH observed in the HE-RG group (6.11) may have impaired passive diffusion across the rumen epithelial membrane, resulting in a lower butyrate absorption rate and greater accumulation in the rumen, resulting in a lower concentration of BHB.

There is evidence of differences in rumen digestion between RFI-divergent cattle [20]. However, this was not observed in the present experiment. In accordance with that, research conducted on beef bulls under a high-concentrate diet (rolled barley, 860 g/kg DM) reported no differences in rumen pH and VFA proportions for divergent RFI groups [21]. On the other hand, a previous study using a diet comprised purely of grass silage [22] reported that HE-RFI cattle tended to have greater ruminal propionate concentrations and lower acetate:propionate ratios. This agrees with results reported by other studies [11,23], also feeding high-fiber diets. A lower acetate:propionate ratio in HE-RFI cattle is consistent with greater energy efficiency and lower methane production. It seems, therefore, that differences in the rumen fermentation profile are evident in high-fiber diets but not in high-concentrate diets, such as those fed to pre-weaning calves.

The concentration of NH3-N was greater in HE-RG calves, in comparison to LE-RG calves, indicating a better rumen fermentation efficiency. In this group, rumen ammonia levels were similar to those observed by previous studies [24]. On the other hand, the LE-RG group presented values below those considered optimal, suggesting a lower ruminal efficiency. This better efficiency in rumen fermentation may have resulted in an increase in microbial protein production and a consequent increase in the flow of metabolizable protein to the intestine. Pre-weaning calves are largely dependent on intestinal digestion for nutrient absorption, and this appears to be the factor responsible for the difference in RG between groups in this current experiment.

In this study, we evaluated the feed efficiency divergence in pre-weaning dairy heifers. In order to produce results that are useful for the dairy industry, we designed an experiment following the feeding practices adopted in commercial dairies (i.e., set amount of milk and ad libitum starter). Although this practice represents common rearing conditions, it limits the understanding of the separate effects of milk and starter. Therefore, it is not possible to determine if the variation observed for certain variables results from milk or starter intake, nor is it possible to study the interactions among them. However, as the milk supply was fixed at 6 L/day for all animals during the trial, the total DMI variation among heifers would be related to the difference in solid feed intake.

## 5. Conclusions

The RFI and RG groups had no negative effects on relevant metabolic and, morphometric measurements or metabolic and performance characteristics. Consequently, such feed-efficiency divergence tests are applicable to the selection of more efficient pre-weaned heifers. However, there was no evidence of strong associations between the blood or rumen metabolites and the RFI and RG tests. In this study, the measurements of metabolic indicators, per se, were not useful in the early identification of more efficient animals. Understanding the underlying physiological basis for improved feed efficiency in dairy heifers requires further investigation.

## Figures and Tables

**Table 1:** Nutrient composition of milk, hay, starter, and the total mixed ration (TMR; 95% starter and 5% hay).

Nutrient Composition (DM Basis, %)	Milk	Hay	Starter	TMR
DM	12.6	90.3	89.3	89.3
CP	3.13	13.6	22.2	21.8
Organic Matter	-	80.8	77.9	78
Ether extract	3.93	3.7	4.6	4.6
NFC	-	16.7	59.5	57.3
NDF	-	70.1	24.5	26.8
ADF	-	33.3	9.9	11
GE (Kcal/kg)	-	3928	3728	3738
Ca	-	0.8	2	1.9
P	-	0.3	0.5	0.5
Lactose	4.54	-	-	-

DM = dry matter; CP = crude protein; NFC = non-fibrous carbohydrate; NDF = neutral detergent fiber; ADF = acid detergent fiber; GE = gross energy; Ca = calcium; P = phosphorus; TMR = total mixed ration.

**Table 2:** Feed intake, feed efficiency (FE), and performance of pre-weaning heifers (4 to 12 weeks of age) classified as high efficiency (HE) and low efficiency (LE, according to residual feed intake (RFI) or residual weight gain (RG).*.

Item	RFI		SEM	*p*-Value			RG		SEM	*p*-Value		
	HE	LE		G	W	G X W	HE	LE		G	W	G X W
DMI (g/day)	1480	1744	51.8	0.06	<0.01	0.94	1535	1594	52.5	0.68	<0.01	0.89
FE	0.7	0.61	0.02	<0.01	<0.01	0.59	0.68	0.63	0.022	0.15	<0.01	0.81
ADG (kg/day)	0.98	0.98	0.03	0.98	<0.01	0.89	1.1	0.92	0.06	0.19	0.37	0.47

* Adapted from a previous study [9]; G = group; W = week and G × W = group by week interaction; FE = feed efficiency (calculated using the ratio between ADG and total DMI).

**Table 3:** Morphometric measurements of pre-weaning heifers (4 to 12 weeks of age) classified as high efficiency (HE) and low efficiency (LE), according to residual feed intake (RFI) or residual weight gain (RG).

Item	RFI		SEM	*p-*Value	RG		SEM	*p-*Value
	HE	LE			HE	LE		
Withers height (cm)								
Initial	79.4	77.9	0.89	0.41	78.9	79.6	1	0.74
Final	94.9	93.2	0.61	0.18	94.1	92.3	0.87	0.31
Variation	15.5	15.3	4.02	0.77	15.2	12.7	0.54	0.01
Hip height (cm)								
Initial	83.3	82	0.99	0.51	83.3	83.4	1.07	0.97
Final	99	97.7	0.67	0.35	97.9	96.7	0.99	0.56
Variation	15.7	15.7	0.57	0.97	14.6	13.3	0.53	0.23
Heart girth (cm)								
Initial	81.2	79.5	0.77	0.28	79.1	80.6	1.02	0.5
Final	106	104	0.98	0.43	104	105	1.14	0.89
Variation	24.8	24.5	0.73	0.94	24.9	24.4	0.86	0.53
Hip width (cm)								
Initial	22.3	20.6	0.41	0.03	20.6	20.3	0.55	0.79
Final	28.6	28.6	0.56	1	29	28.5	0.46	0.6
Variation	6.3	8.0	1.1	0.83	8.4	8.2	0.44	0.83

**Table 4:** Correlation between parameters of morphometric measurements, blood, and ruminal parameters with residual feed intake (RFI) and residual body weight gain (RG).

Item	RFI	RG
**Morphometric measurements**		
Withers height	−0.10	0.01
Hip height	−0.08	0.03
Heart girth	−0.03	0.01
Hip width	−0.05	0.03
**Blood parameters **		
Glucose	0.15	0.61 *
Insulin	0.46 *	0.37
BHB	−0.01	−0.54 *
Glucose:Insulin	0.19	−0.68 *
**Ruminal parameters **		
NH3-N	−0.16	0.42
pH	0.06	0.16
*VFA*	0.03	0.02
Acetate	0.08	−0.13
Propionate	0.13	−0.20
Butyrate	−0.35	0.43

* Asterisk indicates statistical difference in the evaluated correlations (*p* ≤ 0.05).

**Table 5:** Hormones and metabolites of pre-weaning heifers (12 weeks of age) classified as high efficiency (HE) and low efficiency (LE), according to residual feed intake (RFI) or residual weight gain (RG).

Item	RFI		SEM	*p*-Value	RG		SEM	*p*-Value
	HE	LE			HE	LE		
Glucose (mg/dL)	119	112	3.23	0.24	111	107	3.91	0.63
Insulin (μU/mL)	1.72	1.22	0.19	0.18	1.54	1.18	0.257	0.51
BHB (mmol/L)	0.32	0.31	0.15	0.67	0.28	0.38	0.02	0.01
Glucose:Insulin (mg/μU)	0.8	1.17	0.26	0.2	0.83	1.69	0.346	0.07

**Table 6:** Ruminal parameters of pre-weaning dairy heifers (12 weeks of age) classified as high efficiency (HE) and low efficiency (LE), according to residual feed intake (RFI) or residual weight gain (RG).

Item	RFI		SEM	*p-*Value	RG		SEM	*p-*Value
	HE	LE			HE	LE		
NH3-N (mg/dL)	9.87	9.88	1.95	0.99	10.42	6.5	2.27	0.06
pH	5.97	6.24	0.15	0.39	6.11	5.67	0.17	0.21
VFA (µmol/mL)	50.4	41.22	4.23	0.29	51.54	47.23	2.57	0.43
Acetate (µmol/mL)	23.94	20.15	1.93	0.34	21.57	23.14	1.56	0.63
Propionate (µmol/mL)	19.75	17.4	1.87	0.55	18.31	20.14	1.65	0.6
Butyrate (µmol/mL)	3.99	3.32	0.34	0.35	3.99	3.05	0.28	0.1
Acetate:Propionate	1.25	1.2	0.04	0.58	1.19	1.19	0.04	0.95

VFA = volatile fatty acids.

## Data Availability

The data that support the findings and which are presented in this study are available upon reasonable request from the corresponding author, Mariana Magalhães Campos, mariana.campos@embrapa.br. The data is not publicly available, as not all data of the study has been published yet.
